# Cost Considerations in Penile Implantation Revision Surgery from a Global Perspective

**DOI:** 10.1007/s11934-025-01313-0

**Published:** 2026-01-06

**Authors:** Ahmad Majzoub

**Affiliations:** 1 Department of Urology, Hamad Medical Corporation, Doha, Qatar; 2 Department of Clinical Urology, Weill Cornell Medicine -Qatar, Doha, Qatar

**Keywords:** Penile prosthesis implantation, Revision surgery, Erectile dysfunction, Insurance coverage, Healthcare cost

## Abstract

**Purpose of Review:**

Penile prosthesis revision surgery represents a critical yet underexplored aspect of men’s health care. While primary penile prosthesis implantation offers a durable solution for erectile dysfunction unresponsive to medical therapy, complications such as infection, mechanical failure, and patient dissatisfaction often necessitate revision procedures. These surgeries are inherently more complex than primary implantations, requiring greater perioperative resources and incurring higher costs. This review synthesizes global evidence on the economic implications of penile prosthesis revision surgery, with a focus on clinical, procedural, and systemic cost drivers.

**Recent Findings:**

Evidence from both urologic and analogous surgical fields highlights the heightened resource utilization and complication rates associated with revision procedures, particularly in the presence of infection or corporal fibrosis. Device-related considerations, institutional capacity, and healthcare policy play significant roles in determining cost and accessibility. Variations in insurance coverage and access across health systems further underscore disparities in timely and equitable care. Comparative data also demonstrate that revision surgeries carry greater perioperative complexity and higher healthcare expenditure than primary implantations.

**Summary:**

Penile prosthesis revision surgery should be recognized as a distinct and necessary component of sexual medicine services. Risk-adjusted reimbursement models, improved procedural coding, and regionalization of care to high-volume centers are recommended to optimize outcomes and control costs. Addressing disparities in access and ensuring cost-conscious delivery will be essential for advancing equitable and effective care in this field.

## Introduction

Penile prosthesis implantation (PPI) remains a definitive treatment for men with refractory erectile dysfunction (ED) who are unresponsive to pharmacological or other non-invasive therapies. Inflatable penile prostheses, in particular, have demonstrated high patient satisfaction rates, with studies reporting satisfaction levels exceeding 85% [1]. Despite advancements in device design and surgical techniques, complications such as mechanical failure, infection, erosion, or patient dissatisfaction with the functional or cosmetic outcome still persist, necessitating revision surgery [2, 3].

The need for reoperation following PPI is not uncommon. In a population-based study of Medicare beneficiaries undergoing penile prosthesis procedures in California between 2006 and 2009, Grewal et al. (2014) reported an overall reoperation rate of 7.42%, with similar rates between inflatable and semirigid devices [4]. Notably, infectious complications accounted for 3.6% of all cases, while non-infectious failures such as mechanical malfunction were responsible for an additional 4% of reoperations [4]. These findings highlight that both infectious and non-infectious complications contribute substantially to the revision burden, and reoperations may range from straightforward mechanical replacements to more complex procedures involving salvage techniques or management of infected devices which often require greater operative time, specialized surgical expertise, and advanced perioperative care [5, 6].

Despite the increasing attention to value-based surgical care, the economic implications of penile prosthesis revision surgery remain underexplored in the literature. Existing cost analyses primarily focus on primary implantation or on comparing ED treatments more broadly [7, 8]. Revision procedures, though more resource-intensive, are often grouped with general complication costs or inadequately stratified, limiting the precision of economic assessments [9, 10]. This omission prohibits health systems, insurers, and clinicians from making informed decisions about coverage, reimbursement, and cost-effective care pathways.

This review aims to fill the gap by examining the cost considerations in penile prosthesis revision surgery from a global perspective, drawing from available literature, health system reports, and expert consensus. We will explore epidemiology, clinical complexity, direct and indirect costs, and international models that inform sustainable and patient-centered surgical care.

## Clinical and Epidemiological Landscape of Penile Prosthesis Revision: Implications for Cost

**Fig. 1:**
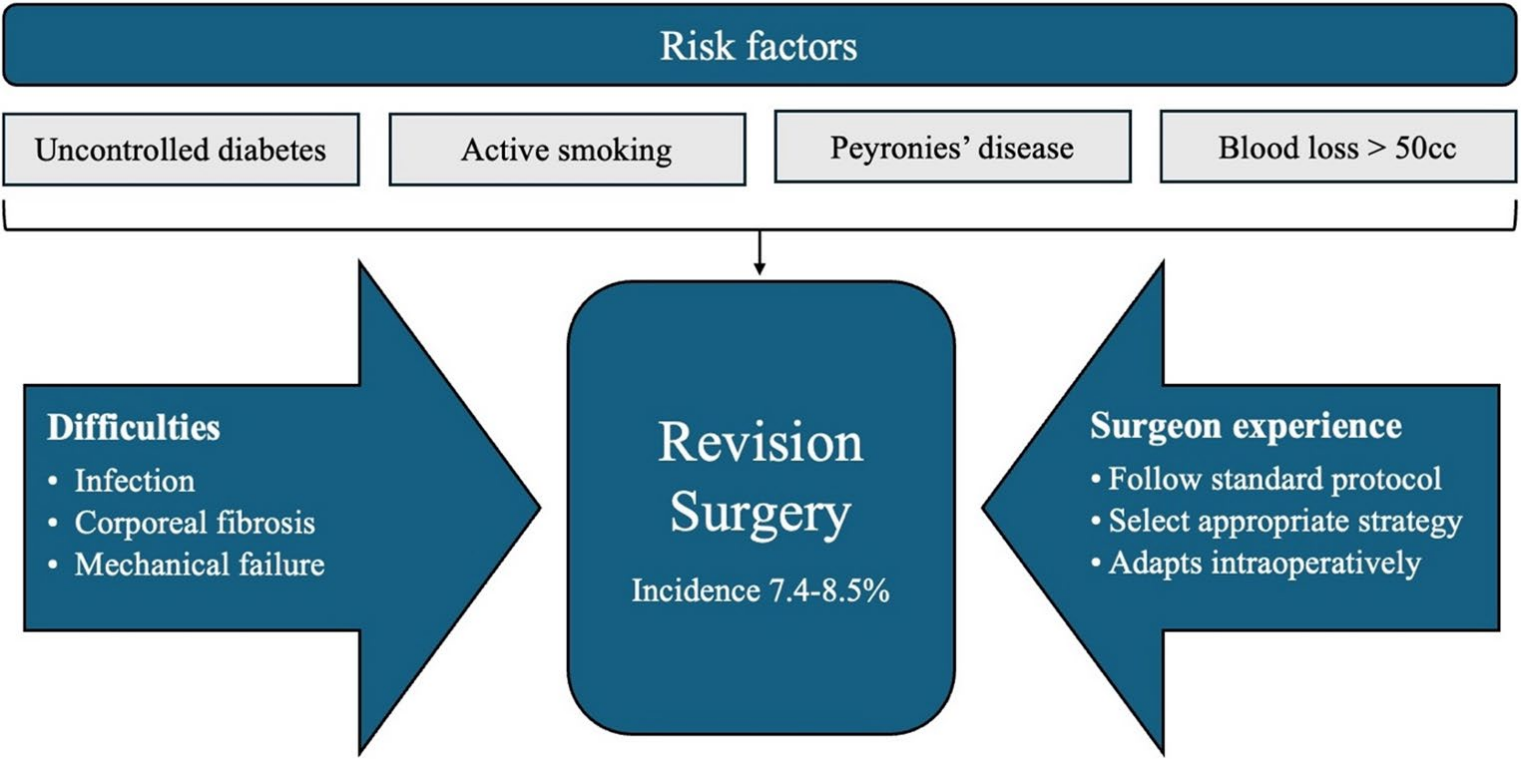
Clinical implications in revision cost

Penile prosthesis revision surgery, while often necessary for restoring sexual function and addressing complications of prior PPIs, may be performed for different reasons and objectives each of which could carry different cost implications. Investigating the indications, patient- and surgeon-related risk factors, and operative complexity of revision procedures is crucial to understanding their economic impact, particularly in light of limited direct cost analysis in the literature (Fig. 1).

As stated earlier, the population-based study by Grewal et al. analyzed 2,263 Medicare beneficiaries who underwent PPI and reported a cumulative reoperation rate of 7.4% at five years, with 3.6% of revisions related to infection and 4.0% due to non-infectious causes such as mechanical malfunction [4]. Similarly, a retrospective analysis conducted at a high-volume Canadian center identified 99 revision procedures among 1,161 implantations (8.5%) between 2006 and 2018. The leading causes were mechanical failure (49.5%), technical complications (28.3%), chronic postoperative pain (12.1%), and infection (10.1%), with a median time to reoperation of 46 months [11]. Device durability remains a key determinant of long-term outcomes; a multicenter study by Wilson et al. estimated a mechanical survival rate of 59.7% at 15 years for inflatable penile prostheses, suggesting a cumulative mechanical failure rate of approximately 40% over this period [12]. These data confirm that the need for penile prosthesis revision persists across both early and late postoperative periods.

Emerging high-quality data underscore several patient-specific factors that significantly elevate the risk of penile prosthesis revision. In a multicenter study by Hawks-Ladds et al. [13], independent predictors for reoperation included uncontrolled diabetes (HbA1c > 8; OR ~ 2.25), active smoking (OR ~ 2.75), Peyronie’s disease (OR ~ 2.47), and intraoperative blood loss exceeding 25 cc (OR ~ 2.45). When focusing specifically on infection-related revisions, the same study identified hypertension (OR ~ 9.12) and use of an infrapubic approach (OR ~ 2.56) as significant risk factors [13]. Most existing literature has concentrated on infection as the primary complication of concern, given its association with high morbidity, the need for re-operation, and ultimately healthcare costs. In a comprehensive systematic review and meta-analysis of 25 studies, Carvajal et al. identified diabetes mellitus as the only statistically significant and consistent predictor of penile prosthesis infection, with a pooled OR of 1.53 (95% CI: 1.15–2.04) [14]. Nonetheless, other comorbidities such as obesity, immunosuppression, and substance use, have been identified as potential risk factors in individual cohort studies, although they did not reach statistical significance in the pooled analyses.

The indication for revision is perhaps the single most important determinant of surgical complexity and resource requirements. Infections, for instance, often necessitate complete device explantation and either delayed reimplantation or immediate salvage. The Mulcahy salvage technique which involves removal of the infected device followed by extensive antibiotic irrigation and immediate placement of a new implant, has been shown to reduce corporal fibrosis and preserve penile length [15]. In a study by Gross et al., 58 patients underwent salvage using malleable prostheses with a mean operative time of 148 min. The salvage procedure was successful in 54 patients (93%), while 4 required explantation due to persistent or recurrent infection. Of the 37 patients who later underwent inflatable reimplantation, 4 (10.8%) experienced new infections requiring removal [16]. These data underscore the elevated reinfection risk and technical burden associated with salvage procedures, especially when transitioning from malleable to inflatable devices.

In addition to infection-related challenges, structural complications such as corporal fibrosis further increase surgical complexity. Corporal fibrosis, frequently the result of prior infection or multiple surgeries, complicates revision surgery by reducing corporal compliance and increasing the risk of intraoperative complications such as urethral perforation or prosthesis misplacement [17]. Management strategies may require advanced techniques including the use of cavernotomes, corporal excavation, or grafting during corporotomy to allow proper cylinder placement [18]. These interventions not only prolong operative time but also require high surgical skill and facility resources, limiting feasibility in low-volume or resource-constrained settings.

Revisions for mechanical failure, although generally performed in uninfected fields, are not without risk. Mechanical malfunction remains a leading indication for revision, with distinct failure patterns between devices, for example tubing fractures are more common in Coloplast implants, while AMS devices show a broader distribution of component failures [19]. Although partial component replacements may be favored for their conservative approach, Amini et al. reported that such revisions were associated with higher rates of complications compared to complete device replacement [20]. In their multicenter analysis, infectious complications occurred in 7.1% of partial revisions versus 2.2% of complete exchanges, while non-infectious complications were reported in 21.2% vs. 9.5%, respectively [20]. Pump-only revisions demonstrated the highest rates of both infectious and non-infectious complications, likely due to retained components harboring biofilm [20]. These findings suggest that even “routine” mechanical revisions warrant careful planning to ensure long-term device integrity and minimize further surgical costs.

In the context of revision surgery, surgeon experience functions as both a clinical and economic determinant. High-volume implanters tend to follow more standardized protocols, select appropriate salvage strategies, and adapt intraoperatively to challenging anatomy, all of which reduce operative time, complication rates, and readmission [21, 22]. Andino et al. found that low-volume surgeons (< 5 implants/year) were significantly more likely to perform revisions due to both mechanical failure and infection, suggesting a correlation between primary implant quality and future revision burden [23]. Recognizing surgeon volume as a modifiable variable offers a pragmatic, systems-level strategy to mitigate the rising cost burden of penile prosthesis care globally.

## Cost Drivers in Revision Surgery

**Fig. 2:**
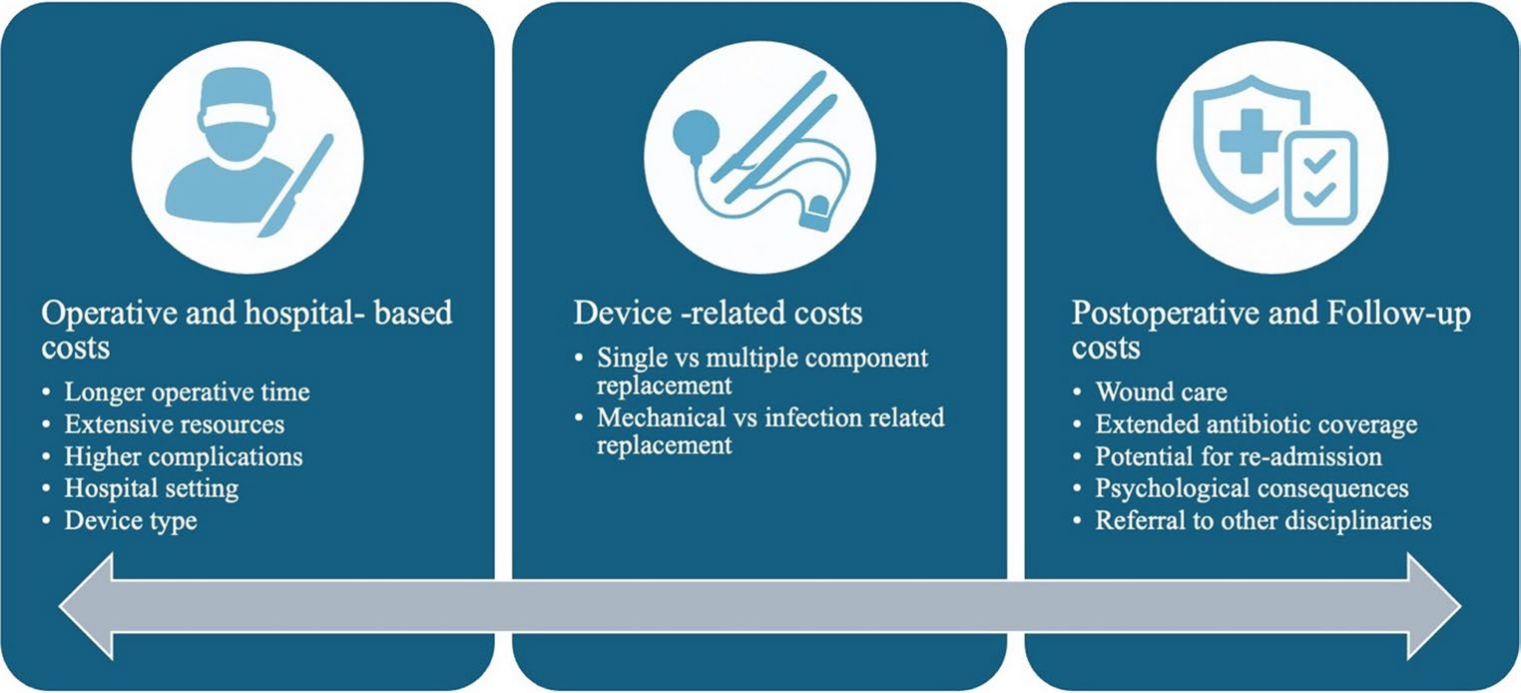
Cost drivers in revision surgery

Penile prosthesis revision procedures are associated with significantly higher costs than primary implantations due to a combination of direct medical expenses, extended operative time, increased resource utilization, and higher complication rates (Fig. 2). While limited studies have specifically quantified the cost of revision surgeries in urology, insights from analogous fields provide a valuable lens through which to understand the economic drivers of these procedures.

### Operative and Hospital-based Costs

Revisions typically require longer operative times and greater intraoperative resources compared to primary procedures. For example, salvage surgery following infection often requires extended operative times exceeding 2.5 h [16]. This increase in duration translates into higher operating room costs, greater anesthesia requirements, and often, prolonged hospital stays. Revision surgeries may also require specialized instruments (e.g., cavernotomes or grafting materials) when dealing with corporal fibrosis, adding to equipment costs.

Similar cost escalations due to revision-related complications have been well documented in other surgical specialties. A 2023 study by van Barreveld et al. examining complications of implantable cardioverter-defibrillators (ICDs) reported an average additional hospital cost of €6,876 per complication, with infections being the most costly at €22,892 per event [24]. Another study by Akindolire et al. assessed 50 cases of 2-stage revisions for periprosthetic joint infection following total hip arthroplasty and found that these infections incurred hospital costs nearly five times higher than uncomplicated primary procedures (Can$38,107 vs. Can$6,764) [25]. Hospital stays were also markedly longer (26.5 vs. 2.0 days), and readmissions were significantly more frequent. These findings reinforce the concept that infection-related complications drive disproportionately high expenditures, regardless of surgical field or device type.

While the majority of cost analyses focus on primary PPI, these data still underscore cost drivers that become magnified in revision contexts. For example, Nguyen et al. demonstrated that out-of-pocket expenses varied significantly based on procedural location (hospital vs. ambulatory center) and prosthesis type (inflatable vs. malleable) [7]. Although their analysis was limited to primary cases, it highlights how factors like operative environment and device selection can substantially influence total cost.

Beyond procedural variables, efforts within the urologic field have begun to focus on predicting and managing economic risk through individualized clinical models. For example, Palmisano et al., in a retrospective analysis of 576 penile prosthesis cases at a Spanish tertiary center, identified revision surgery as an independent predictor of early prosthetic infection (OR: 2.90, *P* = 0.016) [26]. Recognizing the substantial hospital costs associated with treating such infections, the authors developed and internally validated a clinical nomogram that estimates infection risk based on diabetes status, revision history, and implant type. This model can help identify high-risk patients preoperatively, allowing clinicians to implement enhanced infection prevention protocols, consider alternative implant strategies, or refer to high-volume centers when appropriate. By integrating this risk stratification into surgical planning, institutions may reduce the incidence of costly postoperative complications and optimize allocation of hospital-based resources.

### Device-related Costs

The prosthesis itself is among the most expensive components of the procedure. Revision cases often involve full replacement of the implant, though in some cases, only certain components (e.g., pump or cylinders) may be exchanged. However, as the previously referenced study by Amini et al. indicated, partial replacements can paradoxically increase long-term costs due to higher complication rates [20].

Natali et al. compared various prosthesis types in 253 European patients and found that while the AMS 700CX had the highest satisfaction rate (97%), it also had a lower mechanical failure rate and fewer erosions than Ambicor and AMS 600–650 models [27]. Such differences in device performance have cost implications over the long term, particularly in the revision setting. Similarly, Li et al. demonstrated that even mechanical revisions, although often perceived as less complex, were associated with substantial device-related expenses. In their national analysis of inpatient penile prosthesis procedures, the median hospitalization costs ranged from $8,602 to $11,252 depending on the clinical indication [28]. The study also found that early prosthesis removal, particularly during the initial admission, was more frequently observed in non-teaching and low-volume hospitals, as well as among patients with comorbidities such as diabetes, obesity, and chronic renal failure. These findings underscore the importance of both institutional experience and patient-related factors in preventing premature device failure, which ultimately increases the likelihood of repeat procedures and additional prosthesis costs. This further supports the economic rationale for preoperative risk stratification and directing complex cases to high-volume centers with specialized expertise.

In addition, failed salvage procedures or multiple revision attempts may necessitate staged reconstructions or result in long-term device explantation without reimplantation. Each of these outcomes introduces significant additional costs, including the need for repeat anesthesia, advanced imaging, and prolonged follow-up care. These cumulative expenditures highlight how complications related to the device itself can extend well beyond the index procedure and continue to drive costs across the care continuum.

### Postoperative and Follow-up Costs

Postoperative costs extend beyond the operating theater. Patients undergoing revisions are more likely to require close follow-up, wound care, extended antibiotic therapy, and potential re-admissions. Infections, hematomas, or erosions following revision often necessitate unplanned clinic or emergency department visits, further adding to healthcare expenditures [2]. Additionally, the psychological and functional burden of device failure may lead to referrals for counseling or additional treatments, which are not always covered by insurance [29].

Wintner and Lentz emphasize that proactive postoperative management such as structured discharge protocols, nursing follow-up calls, and early complication detection, can significantly reduce unplanned emergency department visits and downstream healthcare use [30]. They also advocate for thorough preoperative counseling to ensure that patients are psychologically prepared for the demands of both the device and the recovery period. In their experience, clear communication of realistic outcomes and a formal follow-up framework minimizes the likelihood of avoidable complications, dissatisfaction, and reactive care, all of which can inflate costs. Huynh et al. further underscore the importance of individualized risk profiling prior to surgery [31]. They review data indicating that poorly controlled diabetes is a strong and quantifiable predictor of postoperative infection, with rates escalating dramatically as HbA1c levels rise: from approximately 1–2% in well-controlled diabetics to 15% when HbA1c reaches 8.6–9.5%, and over 20% when HbA1c exceeds 9.5% [31]. These findings highlight the critical need for metabolic optimization before surgery to prevent infection-related readmissions and costly salvage procedures. Moreover, patients with psychological comorbidities often require more intensive perioperative counseling and post-discharge support. Recent evidence shows that preoperative depression and anxiety are independently associated with higher rates of postoperative complications, infection, and earlier reoperation after IPP surgery [32].

The economic challenges related to postoperative care are echoed in orthopedic literature. In a recent chapter on the financial implications of revision total hip arthroplasty, Ottesen et al. emphasized the burden of prolonged hospitalization, complex equipment requirements, and suboptimal reimbursement models that fail to adequately compensate for the procedural intensity of revision surgery [33]. These observations highlight the need for bundled payment models and cost-containment strategies that are just as applicable in urologic prosthetic surgery.

## Insurance Coverage for Penile Prosthesis Surgery: A Global Perspective

Access to PPI is profoundly influenced by the structure and financing of national healthcare systems. Insurance coverage including both public funding and private reimbursement, determines not only whether patients can undergo implantation but also their ability to access complex revision procedures when needed. This section outlines international variations in insurance policies and access to care, emphasizing the downstream implications of coverage disparities for patients undergoing penile prosthesis surgery (Table 1).

In the United States, PPI is generally reimbursed by Medicare and most private insurance plans when the procedure is deemed medically necessary, typically following failure of conservative medical therapies. Coverage typically includes both the surgical procedure and the prosthetic device itself [34, 35]. However, patient responsibility for deductibles, co-payments, and facility-based charges can be substantial, with out-of-pocket expenses varying widely by insurance plan. Moreover, access remains uneven; patients in rural or under-resourced areas may face geographic barriers, including travel distance to high-volume centers or fellowship-trained surgeons [36]. These disparities may delay care and limit the availability of expertise for complex revision procedures. The differences in clinical complexity are partly acknowledged in the procedural coding structure. Initial IPP placement, device removal, and combined removal-and-replacement are assigned distinct CPT codes with correspondingly different relative value units and reimbursement levels [37]. However, these code-based differentials do not necessarily capture the full economic burden of revision surgery, which also includes increased perioperative risk, higher likelihood of postoperative complications, and greater intensity of follow-up care [38].

In Canada, coverage is determined at the provincial level. While provinces like Alberta provide full reimbursement for prosthesis procedures, others offer partial coverage or exclude the procedure altogether, resulting in out-of-pocket costs of CAD $5,000–$6,000 for some patients [39]. This variation within a universal healthcare framework leads to regional inequities in access, particularly for revision procedures, which may be deprioritized in favor of less complex interventions.

In the United Kingdom, penile prostheses are covered under the National Health Service (NHS) for patients with severe ED unresponsive to other modalities [40]. However, access is constrained by regional commissioning policies and long surgical waitlists. These limitations have led some patients to seek private treatment, where the cost of surgery can exceed £10,000 [41]. Private insurance often excludes penile implants, placing financial responsibility entirely on patients.

Insurance coverage across Europe is heterogeneous. Countries with well-established public healthcare systems, such as France and Germany, generally provide reimbursement for PPI when clinically indicated. For instance, French national hospital data show a 33.8% increase in PPI between 2016 and 2021, indicating sustained public funding and access [42]. In contrast, nations with decentralized insurance frameworks often offer inconsistent coverage or impose restrictions on reimbursement for erectile dysfunction procedures. This has been reflected in studies such as Natali et al., which documented significant variation in prosthesis use, device type selection, and clinical outcomes across European centers suggesting that system-level disparities may influence not only access but also standardization of care [27].

In the Middle East, implant procedures are often covered by government-based healthcare systems for citizens in countries such as Qatar and the UAE [43]. However, expatriates and residents in less wealthy nations face substantial barriers, with limited insurance coverage and out-of-pocket expenses serving as deterrents to care.

Asian countries display a wide range of practices. For example, Singapore’s Medisave program may offer partial reimbursement under specific criteria, while in countries like India or Indonesia, patients frequently self-fund surgeries due to limited public funding and exclusion from most private insurance plans [44–46]. These financial barriers are compounded by shortages of trained implant surgeons and limited device availability.

**Table 1:** Regional variations in insurance coverage for penile prosthesis implantation

Country/Region	Public Coverage	Private Insurance Coverage	Typical Out-of-Pocket Costs	Access Barriers
United States	Yes (Medicare), varies by private insurer	Yes, often with co-pays/deductibles	Moderate to high (varies by plan)	Geographic disparities, insurance gaps
Canada	Provincial variation; some full, others partial/none	Limited; often not covered	CAD $5,000–12,000 (inflatable)	Provincial inequality
United Kingdom	Yes (NHS), but regional restrictions	Rarely covered	Up to £10,000	Long wait times, regional restrictions
France & Germany	Yes, strong public coverage	Varies	Low to none	Lower ED prioritization in some areas
Middle East (Qatar, UAE)	Yes for citizens (government-funded)	Often limited for expatriates	High for non-citizens	Residency status, insurance eligibility
India & Indonesia	No or minimal	Rare	High; mostly self-funded	Cost, device/surgeon availability
Singapore	Partial (Medisave)	Varies by plan	Partial reimbursement under criteria	Coverage criteria, surgeon access

## Policy and Future Directions

The increasing clinical demand and procedural complexity of penile prosthesis revision surgery call for a parallel evolution in health policy and systems-level planning. Current reimbursement models and coding structures often fail to distinguish between primary and revision procedures, leading to inadequate compensation for surgeries that are more technically demanding, resource-intensive, and prone to complications. A critical policy step is the adoption of procedure-specific codes and risk-adjusted reimbursement frameworks that reflect the true cost and complexity of revision surgery.

Regional health authorities and international urological societies should work to reduce disparities in access by investing in surgical training, promoting centralized referral to high-volume centers, and supporting the equitable distribution of prosthetic devices, especially in low- and middle-income countries. While select low-complexity revisions may be safely performed in outpatient settings by experienced surgeons, most cases require inpatient care and advanced perioperative support. Cost-efficient care models must therefore be flexible and tailored to case complexity, rather than relying solely on site-of-service savings.

Incentivizing bundled payment systems that account for revision-specific risks, patient comorbidities, and long-term follow-up needs may help address under-compensation and promote more sustainable care pathways. However, such models must be developed with attention to the unique features of prosthetic surgery, avoiding one-size-fits-all approaches that could inadvertently restrict access to revision procedures.

Future research should prioritize multicenter, prospective evaluations that capture not only the direct costs of revision surgery, but also indirect costs such as lost productivity, reduced quality of life, and the psychosocial consequences of untreated erectile dysfunction. These data are essential to inform shared decision-making, advocate for policy reforms, and support broader payer engagement.

Ultimately, penile prosthesis revision is not simply a re-intervention. It is often a final therapeutic opportunity for patients whose quality of life has been profoundly affected by device failure or explantation. Ensuring access to timely, safe, and appropriately resourced revision care should be recognized as a core objective of sexual medicine policy, both for its functional outcomes and its broader implications for men’s health and well-being.

## Conclusion

Penile prosthesis revision surgery represents a growing and underappreciated component of men’s health care, characterized by high clinical complexity and distinct economic demands. Unlike primary implantation, revision procedures are frequently unavoidable and often represent the only viable option for restoring sexual function after device failure or explantation. Preoperatively, costs are driven by the need for careful risk stratification, metabolic optimization, and psychological support in a population that commonly carries significant comorbidities. Intraoperatively, longer operative times, specialized techniques, and the frequent need for full device replacement increase resource use and amplify the impact of surgeon experience and institutional infrastructure on both outcomes and cost. Postoperatively, higher rates of complications, intensive wound and infection surveillance, and unplanned visits or readmissions further contribute to the overall economic burden. Yet current reimbursement models, coding structures, and access frameworks do not fully reflect these realities. A dedicated focus on procedure-specific coding, centralized expertise, equitable coverage, and real-world cost data is essential to ensure that patients in need of revision receive timely, effective, and financially sustainable care. As the field advances, integrating clinical excellence with economic foresight will be critical to optimizing outcomes and upholding standards of care in sexual medicine.

## Key References

- Hawks-Ladds N, et al. Risk factors for reoperation of inflatable penile prosthesis among an ethnically diverse urban population in a high-volume center. Int J Impot Res. 2025;37(1):37–44.
  - ○ Identifies independent patient- and surgery-related predictors for penile prosthesis reoperation, including uncontrolled diabetes and smoking.
  - ○ Provides robust, contemporary evidence directly relevant to risk stratification and cost modeling in revision surgery.
- Amini E, et al. Malfunction and mechanical failure of the inflatable penile prosthesis: a narrative review of etiologies and management. AME Med J. 2024;9:28–39
  - ○ Synthesizes recent data on mechanical failure patterns and management, highlighting higher complication rates with partial revisions. Informs surgical choices aimed at minimizing long-term costs.
- Andino JJ, et al. Association between surgeon procedure volume and reoperation rates for penile prosthesis implantation. J Sex Med. 2025;22(5):916–923.
  - ○ Demonstrates a strong volume–outcome relationship, with low-volume surgeons significantly more likely to perform revisions.
  - ○ Supports policy changes to regionalize complex cases to high-volume centers.
- Palmisano F, et al. Ten-year experience with penile prosthetic surgery for the treatment of erectile dysfunction: outcomes of a tertiary referral center and predictors of early prosthetic infection. Asian J Androl. 2022;24(1):32–39
  - ○ Identifies revision surgery as an independent predictor of early prosthetic infection and introduces a validated nomogram for infection risk estimation, offering a practical tool for preoperative planning.
- Chang C, et al. New findings regarding predictors of Poor Corporal Integrity in Penile Implant Recipients: A Multicenter International Investigation. BJU Int. 2025;135(3):528–534.
  - ○ Highlights the impact of severe corporal fibrosis on revision surgery complexity, underscoring the importance of specialized techniques and careful resource allocation.

## Data Availability

No datasets were generated or analysed during the current study.
